# Treatment of spontaneous ruptured hepatocellular carcinoma: A single-center study

**DOI:** 10.12669/pjms.303.4001

**Published:** 2014

**Authors:** Hanteng Yang, Kefei Chen, Yongang Wei, Fei Liu, Hongyu Li, Zhipeng Zhou, Bo Li

**Affiliations:** 1 Hanteng Yang, Department of Liver Surgery, West China Hospital, Sichuan University, Chengdu 610041, China.; 2 Kefei Chen, Department of Liver Surgery, West China Hospital, Sichuan University, Chengdu 610041, China.; 3 Yongang Wei, Department of Liver Surgery, West China Hospital, Sichuan University, Chengdu 610041, China.; 4 Fei Liu, Department of Liver Surgery, West China Hospital, Sichuan University, Chengdu 610041, China.; 5 Hongyu Li, Department of Liver Surgery, West China Hospital, Sichuan University, Chengdu 610041, China.; 6 Zhipeng Zhou, Department of Liver Surgery, West China Hospital, Sichuan University, Chengdu 610041, China.; 7 Bo Li, Department of Liver Surgery, West China Hospital, Sichuan University, Chengdu 610041, China.

**Keywords:** Hepatocellular carcinoma, Ruptured, Treatment

## Abstract

***Objectives:*** Spontaneous rupture of hepatocarcinoma (HCC) is a fatal complication of advanced HCC and is associated with poor prognosis. However, there is no consensus on the best approach to treat hemoperitoneum due to ruptured HCC. In this paper, we evaluate and discuss the outcomes of different treatment methods employed at our center for ruptured HCC.

***Methods:*** We reviewed the medical records of 132 patients diagnosed with ruptured HCC at our hospital from January 2003 to December 2012 and evaluated and compared the outcomes of five treatment methods for ruptured HCC: conservative treatment, surgical hemostasis, transarterial embolization (TAE), and one- and two-stage resections.

***Results:*** There was no significant difference in the median survival time between the conservative treatment and surgical hemostasis groups. Patients in the TAE alone group had a better prognosis than those in the conservative treatment and surgical hemostasis groups. The survival time of the tumor resection group was obviously better than that of the conservative treatment, surgical hemostasis, and TAE alone groups, but no significant difference was observed between the one-stage and two-stage resection groups.

***Conclusions:*** One-stage hepatectomy is a better option for patients with preserved liver function, whereas TAE is a better option for those with poorly preserved liver function.

## INTRODUCTION

Hepatocellular carcinoma (HCC) is one of the most common cancers and is associated with a poor prognosis.^1^ It develops in cirrhosis secondary to viral hepatitis or in patients with a history of alcohol abuse.^2^ The incidence of HCC is increasing worldwide with an increasing prevalence of hepatitis C virus infection.^3^^-^^6^ Spontaneous rupture with intraperitoneal hemorrhage is a fatal complication of HCC, having an incidence of 3%–14.5%, which is higher in Asian countries than in western countries.^7^^-^^11^ Although the incidence of ruptured HCC is decreasing because of earlier detection and advanced treatment, the mortality rate within 30 days of HCC rupture remains high at 31%–67%.^1^^,^^10^^,^^12^^-^^16^ However, it is difficult to predict the occurrence of HCC rupture and, consequently, there are a limited number of treatment options, which include conservative treatment, surgical hemostasis, transarterial embolization (TAE), and emergency or staged liver resection. Here we evaluate and compare the outcomes of various treatment methods for ruptured HCC employed at our hospital.

## METHODS

The medical records of 132 patients who were diagnosed with ruptured HCC at our hospital from January 2003 to December 2012 were retrospectively reviewed. Of these 112 (84.8%) males and 20 (15.2%) females; median age, 48.5 (range, 23–78) years, 120 (90.9%) patients had HBV infections and 99 (75%) had liver cirrhosis. Forty-two (31.8%) of the 132 patients were classified as Child–Pugh class A, 52 (39.4%) as class B, and 38 (28.8%) as class C. Twenty-five (18.9%) patients were classified as stage II, as proposed by the Liver Cancer Study Group of Japan,^17^ 41 (31.1%) as stage III, 63 (47.7%) as stage IVA, and 3 (2.3%) as stage IVB. Diagnosis was made on the basis of abdominal paracentesis, ultrasonography, and/or abdominal computed tomography (CT). Shock at the time of admission was defined as a systolic blood pressure <90 mmHg and a pulse rate >100 beats/min. Liver cirrhosis was diagnosed on the basis of the clinical and laboratory examinations and CT or ultrasonography findings consistent with the disease. Data of all patients are summarized in Table-I and their clinical features are presented as medians (range) and frequencies (%). The chi-squared test was used for statistical analysis. The overall survival rate was estimated using the Kaplan–Meier method, and the log-rank test was used to analyze differences in cumulative survival curves. *P* < 0.05 was considered statistically significant. Statistical analyses were performed using SPSS v13.0 (SPSS Inc., Chicago, IL, USA).

## RESULTS

Treatments were based on the patients’ condition at the time of admission, short-term therapeutic effects, and the wishes of the families and included conservative treatment, surgical hemostasis, TAE alone, and one- or two-stage resection. Of the 132 patients, 45 (34.1%) patients received only conservative treatment according to their poor liver function and/or multiple tumor lesion or the wishes of the families, and surgical hemostasis was performed for 8 (13.6%) patients which didn’t maintain hemodynamic stability after conservative management with fluid and blood replacement 24 h of admission because unavailable TAE in early period, which included ligation of the ipsilateral or common hepatic artery, perihepatic packing, and debridement of the ruptured tumor. Tumours in 17(12.9%) patients were considered resectable based on the CT scan and liver function (10 classified as Child–Pugh A and 7 as Child–Pugh B), one-stage tumour resections were carried out within 3 days of admission. Among patients who were haemodynamically unstable (52) underwent conservative management with fluid and blood replacement, 41(31.1%) received only transarterial embolization (TAE) because of either damaged liver function or tumour size, the other 11(8.3%) patients who received TAE were performed two-stage resection at 4–43 days after TAE according to their liver functions (8 classified as Child–Pugh A, 3 as B) and CT scan. The overall median survival time and 30-day and 1-year survival rates were 53 days (range, 1–1012 days), 63.6%, and 17.5%, respectively.

**Fig.1:**
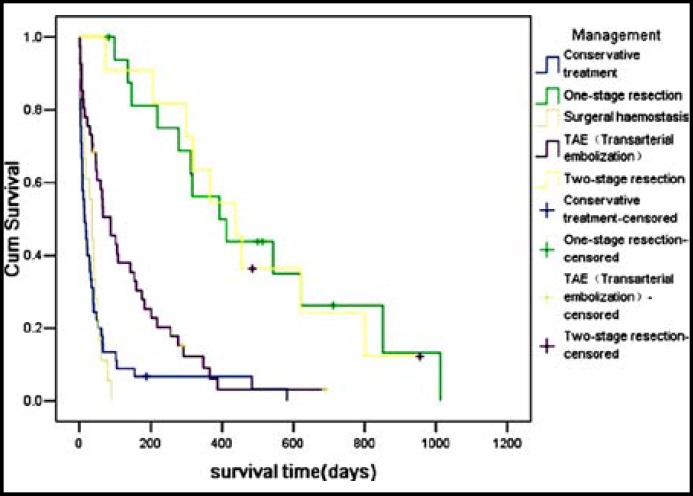
Survival curve of patients underwent different treatment

In the conservative treatment and surgical hemostasis groups, the median survival time and 30-day and 1-year survival rates were 15 days (range, 1–582 days), 35.6%, 6.7% and 35 days (range, 1–89 days), 55.6%, and 0%, respectively. TAE alone was performed in 41 (31.1%) patients; the median survival time and 30-day and 1-year survival rates were 87 days (range, 2–690 days), 73.2%, and 9.1%, respectively. Seventeen (12.9%) patients underwent one-stage resection and 11 (8.3%) underwent two-stage resection; their median survival time and 30-day and 1-year survival rates were 392 days (range, 82–1012 days), 100%, and 56.3%, respectively, and 437 days (range, 74–955 days), 100%, and 63.6%, respectively. The clinical characteristics of the 132 patients who underwent different treatments are shown in Table-II. There was no significant difference in the median survival time between the conservative treatment and the surgical hemostasis groups (*P *> 0.05). The patients who underwent TAE alone had better prognosis than those who underwent conservative treatment and surgical hemostasis (*P* < 0.05). The survival time of the tumor resection group was obviously better than that of conservative treatment, surgical hemostasis, and TAE alone groups (*P* < 0.05), but no significant difference was observed between the one-stage and two-stage resection groups (*P* > 0.05). The survival curves of patients who underwent different treatments are shown in Fig.1.

## DISCUSSION

Hepatocellular carcinoma is often fatal, accounting for approximately 100,000 deaths per year in China ^18^. Spontaneous rupture of HCC is a fatal complication of advanced HCC and is associated with poor prognosis. Reportedly, the 30-day mortality rate of ruptured HCC is 35%–67%. ^10^^, ^^12^^, ^^14^^, ^^19^ In our study, the 30-day mortality rate was 36.4%, consistent with the literature.

Although the precise mechanisms underlying HCC rupture remain unclear, trauma, transarterial chemoembolization, vascular dysfunction in tumor tissues, and portal hypertension have been correlated with HCC rupture.^20^^,^^21^

The primary purpose of treating ruptured HCC is to achieve hemostasis and save patient lives. Although many treatment methods have been reported for ruptured HCC, such as conservative treatment, surgical hemostasis, TAE, and emergency and staged resection, a consensus has not yet been reached on the best approach for treating hemoperitoneum due to ruptured HCC. Patients may present with pre-existing coagulopathy and diminished liver function; therefore, careful pretreatment evaluation is important. Conservative treatment and surgical hemostasis are associated with poor prognosis,^10^^,^^22^ whereas TAE is a feasible treatment option to achieve a good hemostatic effect, but the median overall survival time of TAE alone is limited. ^1^^, ^^16^

**Table-I:** Characteristics of the 132 patients with ruptured HCC

*Variable *	*Total (n = 132)*
Age (years)	48.5 (23–78)
Gender, n (%)	
Male/Female	112 (84.8%)/20 (15.2)
Etiology, n (%)	
HBV/HCV/alcohol/others	120(90.9%)/1(0.8%)/2(1.5%)/9 (6.8)
Liver cirrhosis, n (%)	99 (75%)
Presence of admission, n (%)	
Abdominal pain/Abdominal distension	121 (91.7%)/105 (79.5%)
Child–Pugh class, n (%)	
A/B/C	42(31.8%)/52 (39.4%)/38 (28.8%)
Hypovolemic shock, n (%)	58 (43.9%)
Hemoglobin (g/L)	87.5 (43–128)
Platelet (×10^9^/L)	114.5 (25–365)
Prothrombin time (s)	15.1 (11.6–116.7)
Albumin (g/L)	30 (13.0–44.2)
Total bilirubin (mmol/L)	20.95 (5.9–201)
ALT (IU/L)	56 (18–4291)
Creatinine (μmol/L)	89.1 (44.0–370.6)
AFP (ng/mL)	159.5 (0.62–1511.0)
Thrombosis, n (%)	57 (43.2%)
Ascites, n (%)	90 (67.2%)
Multiple tumors, n (%)	61 (46.2%)
Modified UICC stage (n/%)	
II	25 (18.9%)
III	41 (31.1%)
IVA	63 (47.7%)
IVB	3 (2.3%)
30-day survival rate	63.6%
1-year survival rate	17.5%
Median survival time (days)	53 (1–1012)

***Abbreviations: ***AFP, a-fetoprotein; ALT, alanine aminotransferase; HBV, hepatitis B virus;

HCV, hepatitis C virus; UICC, Union for International Cancer Control.

**Table-II:** Clinical characteristics of the patients with different treatments

*Variable*	*Conservative* *treatment*	*Surgical* *hemostasis*	*TAE only*	*One-stage* *resection*	*Two-stage* *resection*
Number (n/%)	45 (34.1%)	18 (13.6%)	41 (31.1%)	17 (12.9%)	11 (8.3%)
Child–Pugh Classification (n/%)					
A	7 (15.6%)	0	17 (41.5%)	10 (58.8%)	8 (72.7%)
B	13 (28.9%)	12 (66.7%)	17 (41.5%)	7 (41.2%)	3 (27.3)
C	25 (55.6%)	6 (33.3)	7 (17.1%)	0	0
Modified UICC stage (n/%)					
II	1 (0.8%)	0	2 (1.5%)	15 (11.4%)	7 (5.3%)
III	10 (7.6%)	7 (5.3%)	18 (13.6%)	2 (1.5%)	4 (3.0%)
IVA	32 (24.2%)	11 (8.3%)	20 (15.2%)	0	0
IVB	2 (1.5%)	0	1 (0.8%)	0	0
30-day survival rate	35.6%	55.6%	73.2%	100%	100%
1-year survival rate	6.7%	0	9.1%	56.3%	63.6%
Median survival time (days)	15 (1–582)	35 (1–89)	87 (2–690)	392 (82–1012)	437 (74–955)

*Abbreviations:* UICC, Union for International Cancer Control.

Several studies have shown that one-stage liver resection or two-stage hepatectomy has better survival effects compared with other treatment methods. ^6^^, ^^23^^-^^29^ In the present study, the prognosis of conservative treatment and survival hemostasis was poor, and the median survival time was 15 and 35 days, respectively. The median survival time of TAE alone was 87 days, which was better than that of conservative treatment and surgical hemostasis, whereas liver resection showed a better prognosis. The median survival time of one-stage and two-stage resection was 392 and 437 days, respectively, which were superior to the other three treatment methods, but was not statistically significance between themselves. Of course, the patient’s general condition and liver function at the time of admission also affected disease management and prognosis. According to our results, one-stage hepatectomy is recommended for patients with preserved liver function (Child–Pugh classes A and B) and resectable tumors. For patients who cannot tolerate one-stage hepatectomy or for those with unresectable tumors, TAE is a better treatment option, particularly in patients with poorly preserved liver function.

## CONCLUSIONS

The survival time of different treatment methods for ruptured HCC demonstrated that one-stage hepatectomy is a better choice for patients with preserved liver function (Child–Pugh classes A and B) and resectable tumors. For patients who cannot tolerate one-stage hepatectomy or for those with unresectable tumors, TAE is a better treatment option, particularly in patients with poorly preserved liver function. However, the present study was limited by its retrospective nature; therefore, further prospective studies are warranted.

## Author Contribution:

***Bo Li and Yongang Wei:*** Proposed the idea, collected the manuscript and the guarantor.

***Hanteng Yang and CKF:*** Performed search and wrote the first draft.

***Fei Liu, Hongyu Li and Zhipeng Zhou:*** Collected and analyzed the data.

All authors contributed to the design and interpretation of the study and to further drafts.

## Notes

***Funding: ***None declared

## Notes

***Ethical Approval:*** This study was approved by the medical ethics committee of the West China Hospital, Sichuan University.
